# Prevalence of *Clostridium difficile* in raw beef, cow, sheep, goat, camel and buffalo meat in Iran

**DOI:** 10.1186/1471-2458-14-119

**Published:** 2014-02-05

**Authors:** Ebrahim Rahimi, Mohammad Jalali, J Scott Weese

**Affiliations:** 1 Department of Food Hygiene, Faculty of Veterinary Medicine, Shahrekord Branch, Islamic Azad University, P.O. Box: 166 Shahrekord, Iran; 2 Infectious Disease and Tropical Medicine Research Center and School of Food Science and Nutrition, Isfahan University of Medical Sciences, Isfahan, Iran; 3 Department of Pathobiology, Ontario Veterinary College, University of Guelph, Ontario, Canada

**Keywords:** *Clostridium difficile*, Raw meat, Camel, Buffalo, Beef, Antimicrobial resistance

## Abstract

**Background:**

*Clostridium difficile* has been shown to be a nosocomial pathogen associated with diarrhoea and pseudomembranous colitis in hospitalised patients and the infection is believed to be acquired nosocomially. Recent studies have shown the occurrence of *C. difficile* in food animals which may act as a source of infection to humans.The aim of this study was to determine the occurrence of *C. difficile* in retail raw beef, cow, sheep, goat, camel and buffalo meat in Iran.

**Method:**

From April to October 2012, a total of 660 raw meat samples from beef, cow, sheep, goat, camel and buffalo were purchased from 49 butcheries in Isfahan and Khuzestan provinces, Iran, and were evaluated for the presence of *C. difficile* using a method including selective enrichment in *C. difficile* broth, subsequent alcohol shock-treatment and plating onto *C. difficile* selective medium. *C. difficile* isolates were tested for the presence of toxin genes and were typed using PCR ribotyping.

**Results:**

In this study, 13 of 660 meat samples (2%) were contaminated with *C. difficile*. The highest prevalence of *C. difficile* was found in buffalo meat (9%), followed by goat meat (3.3%), beef meat (1.7%), cow (0.94%) and sheep meat (0.9%). Seven of the 13*C. difficile* strains (53.9%) were positive for *tcdA, tcdB* and *cdtB* toxin genes and were classified as ribotype 078. Four strains (30.8%) were positive *tcdA*, and *tcdB*, and one strain (7.7%) was possessed only *tcdB*. The remaining isolate was non-toxigenic. Susceptibilities of 13*C. difficile* isolates were determined for 11 antimicrobial drugs using the disk diffusion assay. Resistance to clindamycin, gentamycin, and nalidixic acid was the most common finding.

**Conclusions:**

To our knowledge, the present study is the first report of the isolation of *C. difficile* from raw buffalo meat. This study indicates the potential importance of food, including buffalo meat, as a source of transmission of *C. difficile* to humans.

## Background

*Clostridium difficile* is a Gram-positive, anaerobic, spore-forming bacterium that has come to the forefront as an important human pathogen. It was initially dismissed as commensal in healthy infants, but was recognized as an important cause of antimicrobial-associated with diarrhoea in the 1970s. It is, now, the most commonly diagnosed cause of antimicrobial-associated and hospital-associated diarrhoea, and the cause of virtually all cases of pseudomembranous colitis [1]. *C. difficile* infection (CDI) more recently was described in non-hospitalized patients without underlying disease or a predisposing risk factor such as antimicrobial exposure, advanced age or significant comorbidities [2,3].

*C. difficile* also appears to be an important cause of enteric disease or a commensal in a wide variety of animal species [4-6]. Food animals are an important source of enteropathogens, and *C. difficile* has been isolated from food animals such as poultry and sheep [4-7], pigs [8,9], chickens, goats and cattle [6] and calves [10]. The types of *C. difficile* found in animals and humans are often indistinguishable [10-12] raising concerns that *C. difficile* might be a zoonotic pathogen [9,11]. In particular, ribotype 078 is commonly found in food animals [5,13] and an increasingly reported cause of community-associated CDI in humans [5,14].

The epidemiology of CDI in Iran is poorly understood. The recent finding of ribotype 078 as the leading ribotype in a small study of CDI in humans in Iran [14] raised concern about the potential for food as a source of infection, but the prevalence of *C. difficile* in food in Iran has never been reported. The aim of this study was to determine the occurrence of *C. difficile* in retail raw beef, cow, sheep, goat, camel and buffalo meat in Iran.

## Methods

### Sample collection

From April to October 2012, a total of 660 raw meat samples from beef (young cattle) (n = 121), cow (adult dairy cow) (n = 106), sheep (n = 150), goat (n = 92), camel (n = 124) and buffalo (n = 67) were purchased unpacked from 49 butcheries in Isfahan and Khuzestan provinces, Iran. These cities are the most prominent national cultural and tourist centers located in the center and south of the country, respectively. From each city 40–55 samples (about 0.5 kg / sample; two sections of meat (10 cm × 10 cm × 3 cm) from neck of each carcasses were aseptically removed) were purchased monthly. All samples were placed in separate sterile plastic bags to prevent from spilling and cross contamination and were immediately transported to the laboratory in a cooler with ice packs and processed within 6 h.

### Isolation and identification of *C. difficile*

The samples were processed immediately upon arrival using aseptic techniques. The detection and isolation method used were based on the method described by Rodriguez-Palacios et al. [15] and de Boer et al. [16]. Briefly, 5 g of each sample was transferred to 20 mL of *C. difficile* broth (CDB; Oxoid SR0048) containing 40 g/l proteose peptone, 5.0 g/l, disodium hydrogen phosphate, 0.1 g/l magnesium sulphate, 2.0 g/l sodium chloride, 6.0 g/lfructose and 1.0 g/l sodium taurocholate supplemented with *C. difficile* selective supplement (Oxoid, UK, Code: SR0173) and 5% (v/v) defibrinated sheep blood. After incubation at 37°C for 10 to 15 days under anaerobic conditions 2 mL of the enrichment broth was added to 2 mL of 96% ethanol in a centrifuge tube and homogenized for 50 min on a shaker at room temperature. After centrifugation (3800 × g for 10 min), a loopful of the sediment was streaked onto *C. difficile* agar base (Oxoid, UK, Code: CM0601) supplemented with an antibiotic supplement for the selective isolation *of C. difficile* (Oxoid, UK, Code: SR0173) and 7% (v/v) defibrinated sheep blood and the plates were incubated for 48 h at 37°C, under anaerobic conditions. Three colonies per plate were subcultured onto tryptone soya agar (Oxoid, UK, Code: CM0131) and tested by standard microbiological and biochemical procedures including odour, Gram stain morphology and L-proline aminopeptidase test [4]. Crudely extracted DNA [boiling method: One colony was suspected in 500 μl distilled water and after heating for 10 min at 95°C, the suspension was centrifuged (5 min, 10000 × g)] was used for PCR confirmation (*tpi* gene detection), determination of toxin gene (*tcdA, tcdB* and *cdtB*), and PCR ribotyping of isolates as performed in previous studies [17,18]. For assurance managed at the lab positive and negative controls were included in each batch.

The limitations of the study include the small number of *C. difficile* isolates which were analysed in the study and the impossibility of sampling from other areas of Iran.

### Antimicrobial susceptibility testing

Antimicrobial susceptibility testing was done by the Kirby–Bauer disc diffusion method using Mueller–Hinton agar (HiMedia Laboratories, Mumbai, India) according to the Clinical Laboratory Standards Institute (CLSI) [19] as has been previously described [4]. The antimicrobial agents tested and their corresponding concentrations were as follows: nalidixic acid (30 μg), ciprofloxacin (5 μg), erythromycin (15 μg), tetracycline (30 μg), doxycycline (30 μg), gentamicin (10 μg), metronidazol (5 μg), ampicillin (10 μg), chloramphenicol (30 μg), vancomycin (30 μg), and clindamycin (2 μg). After incubating the inoculated plate for 48 h at 37°C, under anaerobic conditions, the susceptibility of the *C. difficile* to each antimicrobial agent was measured and the results were interpreted in accordance with interpretive criteria provided by CLSI [19].

## Results and discussion

Table 1 shows the prevalence of *C. difficile* isolated from beef, cow, sheep, goat, camel and buffalo meat in two provinces, Iran. *C. difficile* was isolated 13/660 meat samples (Table 1). There were no significant differences (P > 0.05) in the frequency of positive samples among the meat samples or between Isfahan (4/315, 1.3%) and Khuzestan (9/355, 2.3%) (P > 0.05).

**Table 1:** **Prevalence of *Clostridium difficile *** **detected in beef, cow, sheep, goat, camel and buffalo meat samples in Iran**

**Meat sample**	**No. of samples**	**No. of** ***C. difficile*-positive samples**	**No. of isolates positive for toxins**			**Ribotype 078**
			***tcdA***	***tcdB***	***cdtB***	
Beef	121	2 (1.65%)	2	2	2	2
Cow	106	1 (0.94%)	1	1	-	-
Sheep	150	1 (0.67%)	1	1	0	-
Goat	92	3 (3.26%)	2	2	2	2
Buffalo	67	6 (8.96%)	5	6	3	3
Camel	124	0 (0.00%)	-	-	-	-
Total	660	13 (1.97%)	11	12	7	7

The highest prevalence of *C. difficile* was found in buffalo meat samples (6/67), followed by goat (3/92), beef (2/121) and sheep (1/150) (Table 1). All the camel meat samples found to be negative. Toxigenic *C. difficile* strains (*tcdA, tcdB* and *cdtB* toxin genes) were detected in 12/13 of isolates. Seven of the 13*C. difficile* strains (53.9%) were positive for*tcdA, tcdB* and *cdtB* toxin genes. Four strains (30.8%) were positive *tcdA*, and *tcdB*, and one strain (7.7%) was possessed only *tcdB*. The remaining isolate was non-toxigenic. Our finding of *C. difficile* and its toxigenic strains in meat are supported by similar reports from other counters [9,15,16,20-23]. The low prevalence of *C. difficile* in cow, beef, goat and sheep meat samples are comparable with those reported by others [16,20-23]. However, higher contamination rates (20% to 50%) have also been reported [9,15]. In contrast, Von Abercron et al. [24] did not detect *C. difficile* in meat samples other than beef. Whether this reflects a true different prevalence or is due to differences in sampling techniques employed (meat sample, carcass swab or carcass rinse fluid sample), seasonal effects [20] and/or laboratory methodologies employed in different studies is not clear.

The source of *C. difficile* in food products is unclear. Contamination of meat might be due to *C. difficile* residing in the gastrointestinal tract of animals, but could also originate from the hands of personnel working in the slaughterhouse, meat processing equipment or the slaughterhouse environment during the slaughtering process [5,25,26]. The prolonged survival of *C. difficile* spores in the environment increases the possibilities for contamination of animals and foods. Another potential source of infection that requires investigation is the presence of *C. difficile* spores in healthy muscle tissue in living animals [1].

Seven of the 13*C. difficile* strains were positive for *tcdA, tcdB* and *cdtB* toxin genes and were classified as ribotype 078. The predominance of ribotype 078 is consistent with other studies of food animals and food [8,23,27]. Given the presence of this strain in humans in the same region with CDI, consideration must be given as to whether food might be the source. However, further study is required to determine whether food is a reasonable source of infection.

Antimicrobial susceptibility data are presented in Table 2. Resistance of *C. difficile* to clindomycin, gentamycin, nalidixic acid, ciprofloxacin, erythromycin, ampicillin, and tetracycline was high. These results are comparable to those reported by other investigators [6,22,28]. All the *C. difficile* isolates were susceptible to metronidazole, and vancomycin as was observed in other studies [6,15,22]. These two drugs are the most commonly used to treat *C. difficile* diarrhea in humans but are not used in food animals. The results of antimicrobial resistance found in this study are correlated with antibiotics usage to treat infections in food animals in Iran. In contrast, many of the drugs to which the isolates were resistant (i.e. gentamicin) are commonly used in food animals.

**Table 2:** **Antimicrobial resistance of 13 *Clostridium difficile *** **isolated from beef, cow, sheep, goat, camel and buffalo meat in, Iran**

**Antimicrobial agent**	**Sensitive**	**Intermediate**	**Resistant**
Ampicillin	3 (23.08%)	3 (23.08%)	7 (53.85%)
Chloramphenicol	11 (84.62%)	2 (15.38%)	0 (0.0%)
Ciprofloxacin	1 (7.69%)	2 (15.38%)	10 (76.92%)
Clindamycin	0 (0.0%)	1 (7.69%)	12 (92.31%)
Doxycycline	10 (76.92%)	3 (23.08%)	0 (0.0%)
Erythromycin	3 (23.08%)	2 (15.38%)	8 (61.54%)
Gentamicin	0 (0.0%)	0 (0.0%)	13 (100%)
Metronidazole	13 (100%)	0 (0.0%)	0 (0.0%)
Nalidixic acid	0 (0.0%)	0 (0.0%)	13 (100%)
Tetracycline	5 (38.46%)	4 (30.77%)	4 (30.77%)
Vancomycin	13 (100%)	0 (0.0%)	0 (0.0%)

## Conclusions

This study indicates that the potential importance of food, including buffalo meat, as a source of transmission of *C. difficile* to humans. Slaughterhouses can be heavily contaminated with foodborn pathogens [29-31], the maintenance of slaughter hygiene, regular microbiological monitoring of carcasses, implementation of good manufacturing practices and a food safety system such as the HACCP system are essential to minimize the risk to the consumer. To the author’s knowledge, the present study is the first report of the isolation of *C. difficile* from raw beef, cow, goat, sheepand buffalo meat in Iran. Further studies are required to determine the prevalence of *C. difficile* in meat in Iran and to explore the potential risk of human infection with *C. difficile* via consumption of meat.

## Abbreviations

C. difficile: *Clostridium difficile*; CDI: *C. difficile* infection; PCR: Polymerase chain reaction; CLSI: Clinical laboratory standards institute.

## Competing interests

The authors declare that they have no competing interests.

## Authors’ contributions

The supporting of project were performed by ER, MJ and JSW, DNA extraction, PCR techniques were performed by ER and MJ and samples collection, culture, statistical analysis and writing of manuscript were performed by ER. All authors read and approved the final manuscript.

## Pre-publication history

The pre-publication history for this paper can be accessed here:

http://www.biomedcentral.com/1471-2458/14/119/prepub

## References

[B1] Weese JS *Clostridium difficile* in food-innocent bystander or serious threat? Clin Microbiol Infect 2010 16 3 10 20002685 10.1111/j.1469-0691.2009.03108.x

[B2] Bauer MP Veenendaal D Verhoef L Bloembergen P van Dissel JT Kuijper EJ Clinical and microbiological characteristics of community-onset *Clostridium difficile* infection in The Netherlands Clin Microbiol Infect 2009 15 1087 1092 10.1111/j.1469-0691.2009.02853.x 19624512

[B3] Wilcox MH Mooney L Bendall R Settle CD Fawley WN A case–control study of community-associated *Clostridium difficile* infection J Antimicrob Chemother 2008 62 388 396 10.1093/jac/dkn163 18434341

[B4] Harvey RB Norman KN Andrews K Hume ME Scanlan CM Callaway TR Anderson RC Nisbet DJ *Clostridium difficile* in poultry and poultry meat Foodborne Pathog Dis 2011 8 1321 1323 10.1089/fpd.2011.0936 21877928

[B5] Keessen EC Gaastra W Lipman LJA *Clostridium difficile* infection in humans and animals, differences and similarities Vet Microbiol 2011 153 205 217 10.1016/j.vetmic.2011.03.020 21530110

[B6] Simango C Mwakurudza S *Clostridium difficile* in broiler chickens sold at market places in Zimbabwe and their antimicrobial susceptibility Int J Food Microbiol 2008 124 268 270 10.1016/j.ijfoodmicro.2008.03.020 18448182

[B7] Al Saif N Brazier JS The distribution of *Clostridium difficile* in the environment of South Wales J Med Microbiol 1996 45 133 137 10.1099/00222615-45-2-133 8683549

[B8] Songer JG Trinh HT Killgore GE Thompson AD McDonald LC Limbago BM *Clostridium difficile* in retail meat products, USA 2007 Emerg Infect Dis 2009 15 819 821 10.3201/eid1505.081071 19402980 PMC2687047

[B9] Songer JG *Clostridia* as agents of zoonotic disease Vet Microbiol 2010 140 399 404 10.1016/j.vetmic.2009.07.003 19682805

[B10] Rodriguez-Palacios A Stämpfli HR Duffield T Peregrine AS Trotz-Williams LA Arroyo LG Brazier JS Weese JS *Clostridium difficile* PCR ribotypes in calves, Canada Emerg Infect Dis 2006 12 1730 1736 10.3201/eid1211.051581 17283624 PMC3372327

[B11] Goorhuis A Debast SB Van Leengoed LA Harmanus C Notermans DW Bergwerff AA Kuijper EJ *Clostridium difficile* PCR ribotype 078: an emerging strain in humans and in pigs? J ClinMicrobiol 2008 46 1157 1158 10.1128/JCM.01536-07 PMC2268365 18326836

[B12] Rupnik M Wilcox MH Gerding DN *Clostridium difficile* infection: new developments in epidemiology and pathogenesis Natur Rev Microbiol 2009 7 526 536 10.1038/nrmicro2164 19528959

[B13] Pirs T Ocepek M Rupnik M Isolation of *Clostridium difficile* from food animals in Slovenia J Med Microbiol 2008 57 790 792 10.1099/jmm.0.47669-0 18480339

[B14] Jalali M Khorvash F Warriner K Weese JS *Clostridium difficile* infection in an Iranian hospital BMC Res Notes 2012 5 159 10.1186/1756-0500-5-159 10.1186/1756-0500-5-159 22436392 PMC3317812

[B15] Rodriguez-Palacios A Staempfli HR Duffield T Weese JS *Clostridium difficile* in retail ground meat Canada Emerg Infect Dis 2007 13 485 487 10.3201/eid1303.060988 17552108 PMC2725909

[B16] de Boer E Zwartkruis-Nahuis A Heuvelink AE Harmanus C Kuijper EJ Prevalence of *Clostridium difficile* in retailed meat in The Netherlands Int J Food Microbiol 2011 144 561 564 10.1016/j.ijfoodmicro.2010.11.007 21131085

[B17] Bidet P Barbut F Lalande V Burghoffer B Petit J Development of a new PCR-ribotyping method for *Clostridium difficile* based on ribosomal RNA gene sequencing FEMS Microbiol Lett 1999 175 261 266 10.1111/j.1574-6968.1999.tb13629.x 10386377

[B18] Lemee L Dhalluin A Testelin S Mattrat M Maillard K Lemeland J Pons JL Multiplex PCR targeting *tpi* (triose phosphate isomerase), *tcdA* (Toxin A), and *tcdB* (Toxin B) genes for toxigenic culture of *Clostridium difficile* J Clin Microbiol 2004 42 5710 5714 10.1128/JCM.42.12.5710-5714.2004 15583303 PMC535266

[B19] Clinical and Laboratory Standards Institute (CLSI) Performance Standards for Antimicrobial Susceptibility Testing; 7th ed, CLSI document M11–A7 2007 Wayne, PA: Clinical and Laboratory Standards Institute

[B20] Rodriguez-Palacios A Reid-Smith RJ Staempfli HR Daignault D Janecko N Avery BP Martin H Thompson AD McDonald LC Limbago B Weese JS Possibility of seasonality of *Clostridium difficile* in retail meat, Canada Emerg Infect Dis 2009 15 802 805 10.3201/eid1505.081084 19402975 PMC2687045

[B21] Bouttier S Barc MC Felix B Lambert S Collignon A Barbut F *Clostridium difficile* in ground meat, France Emerg Infect Dis 2010 16 733 735 10.3201/eid1604.091138 20350408 PMC3321948

[B22] Jöbstl M Heuberger S Indra A Nepf R Köfer J Wagner M *Clostridium difficile* in raw products of animal origin Int J Food Microbiol 2010 138 172 175 10.1016/j.ijfoodmicro.2009.12.022 20079946

[B23] Curry SR Marsh JW Schlackman JL Harrison LH Prevalence of *Clostridium difficile*in uncooked ground meat products from Pittsburgh, Pennsylvania Appl Environ Microbiol 2012 78 4183 4186 10.1128/AEM.00842-12 22504814 PMC3370506

[B24] Von Abercron SMM Karlsson F Wigh GT Wierup M Krovacek K Low occurrence of *Clostridium difficile* in retail ground meat in Sweden J Food Prot 2009 72 1732 1734 19722410 10.4315/0362-028x-72.8.1732

[B25] Baker AA Davis E Rehberger T Rosener D Prevalence and diversity of toxigenic *Clostridium perfringens* and *Clostridium difficile* among swine herds in the midwest Appl Environ Microbiol 2010 76 2961 2967 10.1128/AEM.02459-09 20208029 PMC2863449

[B26] Thitaram SN Frank JF Lyon SA Siragusa GR Bailey JS Lombard JE Haley CA Wagner BA Dargatz DA Fedorka-Cray PJ *Clostridium difficile* from healthy food animals: optimized isolation and prevalence J Food Prot 2011 74 130 133 10.4315/0362-028X.JFP-10-229 21219775

[B27] Avbersek J Janezic S Pate M Rupnik M Zidaric V Logar K Vengust M Zemljic M Pirs T Ocepek M Diversity of *Clostridium difficile* in pigs and other animals in Slovenia Anaerobe 2009 15 252 255 10.1016/j.anaerobe.2009.07.004 19632350

[B28] Indra A Schmid D Huhulescu S Hell M Gattringer R Hasenberger P Fiedler A Wewalka G Allerberger F Characterization of clinical *Clostridium difficile* isolates by PCR ribotyping and detection of toxin genes in Austria, 2006–2007 J Med Microbiol 2008 57 702 708 10.1099/jmm.0.47476-0 18480326

[B29] Rahimi E Momtaz H Bonyadian M PCR detection of *Campylobacter* sp. from turkey carcasses during processing plant in Iran Food Control 2010 21 692 694 10.1016/j.foodcont.2009.10.009

[B30] Rahimi E Momtaz H Ameri M Ghasemian Safai H Ali Kazemi M Prevalence and antimicrobial resistance of *Campylobacter* species isolated from chicken carcasses during processing in Iran Poult Sci 2010 89 1015 1020 10.3382/ps.2009-00090 20371855

[B31] Rahimi E Momtaz H Nozarpour N Prevalence of *Listeria* spp., *Campylobacter* spp., and *Escherichia coli* O157:H7 isolated from camel during processing BJVM 2010 13 179 185

